# Detection of SARS-CoV-2 RNA in wastewater from an enclosed college campus serves as an early warning surveillance system

**DOI:** 10.1371/journal.pone.0288808

**Published:** 2023-07-20

**Authors:** Jacob Kazenelson, Tori Jefferson, Ryan G. Rhodes, Lawrence B. Cahoon, Arthur R. Frampton

**Affiliations:** Department of Biology and Marine Biology, University of North Carolina Wilmington, Wilmington, NC, United States of America; University of Pécs: Pecsi Tudomanyegyetem, HUNGARY

## Abstract

SARS-CoV-2, the causative agent of Covid-19, is shed from infected persons in respiratory droplets, feces, and urine. Using quantitative PCR (qPCR), our group hypothesized that we could detect SARS-CoV-2 in wastewater samples collected on a university campus prior to the detection of the virus in individuals on campus. Wastewater samples were collected 3 times a week from 5 locations on the main campus of the University of North Carolina Wilmington (UNCW) from July 24, 2020 to December 21, 2020. Post-collection, total RNA was extracted and SARS-CoV-2 RNA in the samples was detected by qPCR. SARS-CoV-2 signal was detected on campus beginning on August 19 as classes began and the signal increased in both intensity and breadth as the Fall semester progressed. A comparison of two RNA extraction methods from wastewater showed that SARS-CoV-2 was detected more frequently on filter samples versus the direct extracts. Aligning our wastewater data with the reported SARS-CoV-2 cases on the campus Covid-19 dashboard showed the virus signal was routinely detected in the wastewater prior to clusters of individual cases being reported. These data support the testing of wastewater for the presence of SARS-CoV-2 and may be used as part of a surveillance program for detecting the virus in a community prior to an outbreak occurring and could ultimately be incorporated with other SARS-CoV-2 metrics to better inform public health enabling a quick response to contain or mitigating spread of the virus.

## Introduction

Screening wastewater has provided data about the presence of various substances, including pathogens, that are excreted in feces, urine, and saliva [1]. Wastewater based epidemiology (WBE) techniques have been successfully used to identify, quantitate, track, and estimate the prevalence of a wide range of viral and bacterial pathogens, as well as environmental contaminants including heavy metals and pharmaceuticals [2–7]. Recently, multiple groups have deployed WBE methodologies to better understand the dynamics of the SARS-CoV-2 pandemic [8–11]. SARS-CoV-2 is the etiologic agent of Covid-19 and this novel coronavirus, first reported in December 2019 in Wuhan China, has rapidly spread around the world. As of this writing, over 5 million people have died after infection with SARS-CoV-2 including one million Americans according to the World Health Organization{World, 2021, WHO Coronavirus (COVID-19) Dashboard}. While the primary route of transmission is via respiratory droplets, the virus is also shed in high quantities in feces and urine [1]. The excretion of SARS-CoV-2 into local sewer sheds provides scientists with new ways to detect and quantitate the amount of virus that is present in a community [12, 13]. Screening wastewater has many advantages over individual testing including anonymity, lower cost, and early detection before the virus spreads widely in a community.

Previous research showed that SARS-CoV-2 RNA can be reliably detected and quantitated in wastewater samples using quantitative PCR (qPCR) and newer PCR technology including droplet digital PCR (ddPCR) [14]. The detection of pathogens in clinical isolates using nucleic acid-based detection methods is well established and yields consistent and reliable results. However, in contrast to clinical specimens, wastewater samples require more extensive upstream processing before the samples are ready to be tested. To this end, many different sampling and processing methods are being compared to determine which methods provide sufficient yields of high-quality RNA that can be accurately detected and quantified using PCR. Methods for sampling include collecting grab samples or using automated samplers to collect the wastewater. After collection, a variety of methods have been employed to process these samples. Two processing methods include filtration on membranes and direct extraction of the samples prior to testing. Both methods have been shown to be effective and each has been used to detect microbial genetic sequences found in wastewater [14]. We compared these sampling methodologies to ascertain the most effective technique.

## Materials and methods

### Sample collection

**Fig 1:**
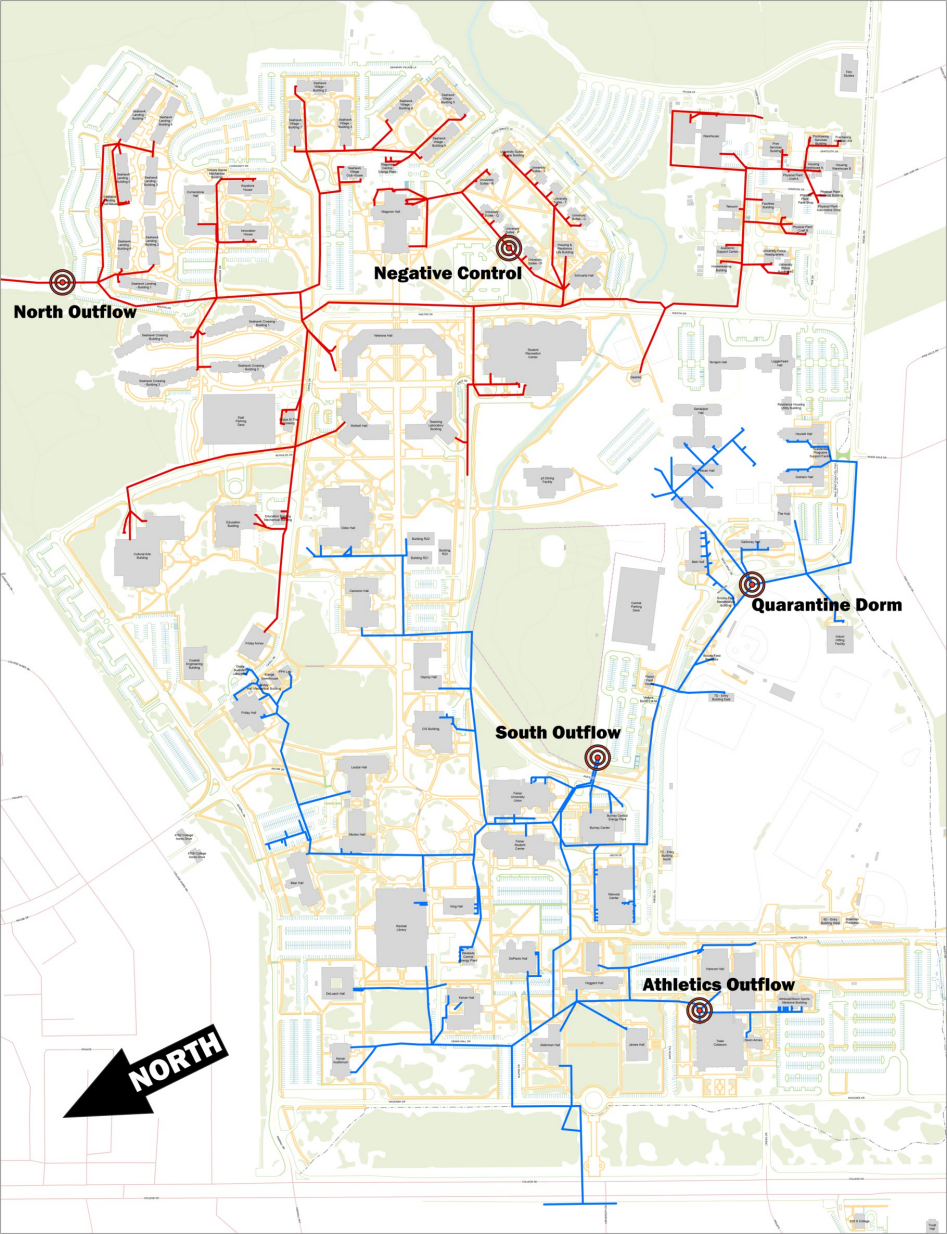
Map of UNCW campus displaying sample sites, sewer system and flow separation. The five targeted sites shown are the physical sample site locations. The northern residential side is lined in red, while the southern “academic” side of campus is lined blue.

Our study was conducted on the main campus of the University of North Carolina Wilmington (UNCW) (Fig 1). The campus comprises 661 acres and, during a typical academic year, approximately 17,500 students and 2,100 employees learn and work on campus. In Fall 2020, 3685 students resided on campus at the start of the 2020–2021 academic year.

The UNCW campus has a secondary wastewater treatment system that is primarily gravity fed with two pump stations servicing the lower levels and a 16 MGD capacity. The system has two major access points that feed into the Cape Fear Public Utility Authority’s wastewater treatment plants. For our study, wastewater grab samples were collected between 8–9 AM local time from 5 separate locations on campus (Fig 1) beginning on July 24, 2020 and ending on December 21, 2020. Two of the sample locations serve as major access points, each collecting wastewater from approximately half of campus. Sample site 1 (North Outflow) collects wastewater from the East-northeastern side of campus and sample site 2 (South Outflow) collects from the south-southwestern side. In addition to dormitories, each of these major sites (Northern and Southern outflow) also collect from administrative and classroom buildings. The other 3 sample sites include the athletics outflow, an empty dorm that served as a negative control, and the quarantine dormitory. Wastewater sampled from the empty dorm was as a result of 2x flushing of the dormitory single occupancy toilet every sampling event performed by plumbing staff.

Collection of wastewater during the summer of 2020 enabled us to establish a baseline prior to students returning for the academic year. From July 24 to August 14 a weekly grab sample was collected from all 5 sites. Starting on August 19, grab samples were collected three times per week from each of the 5 sites and this sampling effort continued until the end of the study on December 21, 2020. In all, a total of 220 samples were collected.

Each sample was collected in a 1-liter mason jar and the contents were transferred to a 250mL polypropylene bottle. Bottles were sprayed with 95% ethanol prior to placing in a cooler and transporting to the laboratory for processing.

### Sample processing

Samples were processed using two methodologies: direct extracts (DE) and membrane filtration (MF) using a 0.45μM 47mm HA filter (Millipore, Burlington, MA). Prior to processing, all sample bottles were pasteurized in a 75°C water bath for 40 minutes. Following heat inactivation, each sample bottle was mixed by inversion, and for direct extracts 600 μL was removed and added to an equal volume of RNeasy RLT Plus lysis buffer (Qiagen, Valencia, CA) in a microcentrifuge tube. Duplicate direct extracts were made for each site prior to storage at -80°C. The process of direct extraction was approximately 45 minutes shorter than membrane filtration. Total time from sample gathering to qPCR results sharing with interested parties was approximately 5 hours utilizing the membrane filtration technique.

Filtration samples were vacuum filtered through a 0.45μM 47mm HA filter using a Millipore stainless steel vacuum filtration apparatus. The volume of sample filtered varied from 10 mL to 100 mL depending upon turbidity. After filtration, each membrane was placed in a microcentrifuge tube containing 850 μL of RNeasy RLT Plus lysis buffer. Duplicate filtration samples were made for each site prior to storage at -80°C.

### RNA extraction

Total RNA from the samples collected by both methods (DE and MF) was extracted using an RNeasy Plus mini kit (Qiagen) per the manufacturer’s instructions. The elution step was repeated to obtain a total elution volume of 100 μL. RNA was used immediately for RT-qPCR and the remaining sample was stored at -80°C.

### RT-qPCR

N1 and N2 primers, which bind to two separate sequences within the SARS-CoV-2 nucleocapsid (N) gene, and the corresponding probes were purchased from Integrated DNA Technologies (IDT, Coralville, IA) and were manufactured under the EUA research guidelines with primer sequences for the novel SARS CoV-2 virus provided from the United States Centers for Disease Control and Prevention [15]. PCR reactions were conducted using BIO-RAD Universal Probes One-Step kit (BIO-RAD, Hercules, CA) according to the manufacturer’s instructions. The N-Plasmid positive control (IDT, Coralville, IA) was used at 1,000 copies per reaction for both N1 and N2. Additionally, a no template control was performed in duplicate for each reaction to ensure no contamination.

Reactions were placed in the BIO-RAD CFX 96 thermal cycler and run under the following program: 10 minutes at 50°C, 3 minutes at 95°C, followed by 43 cycles of 95°C for 15s then 58°C for 30 seconds with fluorescence collected at the end of each cycle. Data was analyzed using the CFX Maestro data analysis software (BIO-RAD).

### Covid-19 health data

The daily, confirmed, SARS-CoV-2 positive individuals’ data were provided by the UNCW dashboard reporting system: https://uncw.edu/bestnest/datadashboard.html. This dashboard also tracked the number of tests administered by both type (molecular, antigen, or antibody) and reason (symptomatic/contact tracing, surveillance: athletics-related, and surveillance: non-athletics-related). In addition, the number of quarantine beds in use at the designated quarantine dormitory was tracked daily. The university was unwilling to utilize WBE data to inform nasal testing and so all data was correlated with only publicly available case numbers published on their dashboard.

### Data analysis

All statistical analyses were performed using JMP Pro v. 16 (SAS Institute, Cary, NC). Student’s t-test was used to determine any significant difference overall between values of the two primer sets (N1 versus N2). One-way ANOVA’s were performed to determine significant differences in Ct values of N1 or N2 vs. Location, followed by a comparison of means using Tukey-Kramer HSD. Linear regression was used to determine the impact of lower or higher Ct values on the difference between these two primer sets by testing for equal slopes. P-values < 0.05 were considered statistically significant for all tests performed. To determine if there was an effect of location on N1 minus N2 Ct values, we used one-way ANOVA.

## Results

### SARS-CoV-2 RNA detection in wastewater samples

**Fig 2:**
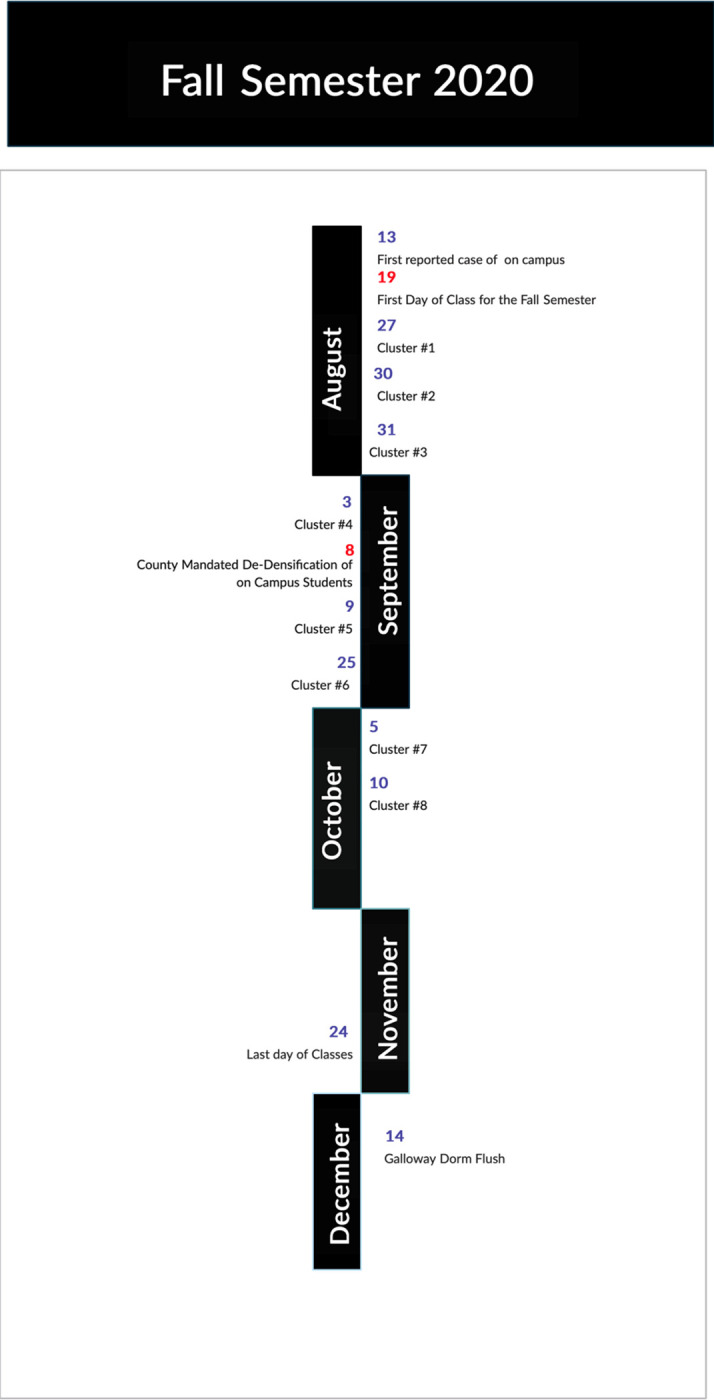
A timeline of SARS CoV-2 infection clusters and events on UNCW’s campus during the fall semester 2020. Dates in red are campus related events.

A timeline of UNCW campus calendar events, including student move in weekend, the start date for fall classes, the first reports of individual SARS-CoV-2 positive cases on campus, the New Hanover County mandated de-densification process, and the announcements of SARS-CoV-2 positive clusters, is shown in Fig 2. A total of eight SARS-CoV-2 clusters were publicly announced by the university during the Fall 2020 semester (Fig 2). Clusters are defined by the North Carolina Department of Health and Human Services [16] as five or more cases that are considered in close proximity by location in the last 14 days.

**Fig 3:**
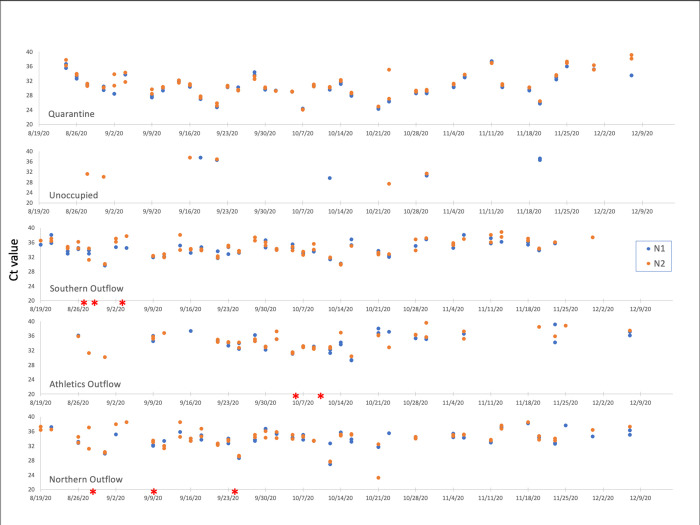
Ct values separated by site and primer set during fall semester 2020. The blue dots represent signal from the N1 primer set, and the orange dots represent signal from N2. The red asterisks * indicate an individual cluster outbreak and are shown on the graph for the location they occurred in, they do not reflect Ct values. Note, infected residential students were moved to the quarantine dorm. Exact cluster dates can be found in Fig 2.

**Fig 4:**
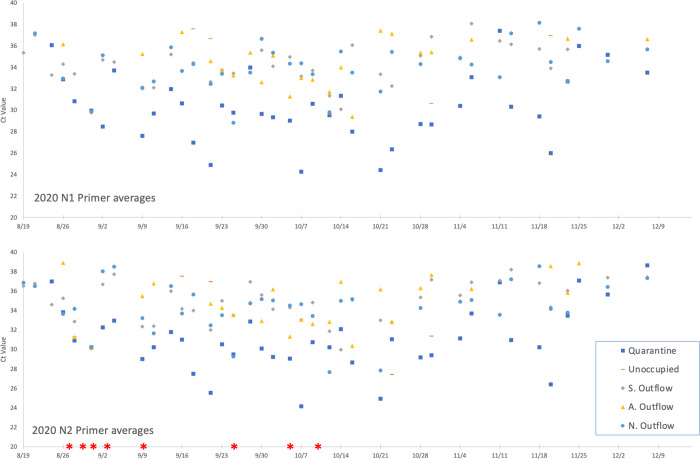
N1 and N2 primer Ct averages by site during fall 2020 semester. Ct values only displayed if there was more than one positive value. Legend displays the 5 sites and the red asterisks * shown at the bottom of the figure are the individual cluster outbreaks and do not reflect Ct.

A wastewater sample was considered positive when one or both nucleocapsid sequences (N1 and N2) were detected by PCR in duplicate. When our sampling effort began on July 24, 2020, the university campus was mostly uninhabited, as all summer classes were delivered online, only a few students resided on campus, and only essential workers were present in person. PCR cycle threshold (Ct) values for both N1 and N2 primer sets are plotted by location over time in Fig 3 and the Ct values for each primer set at all locations are plotted individually over time in Fig 4. The samples collected from all 5 sites on July 24 were negative for SARS-CoV-2 RNA (Figs 3 & 4). Interestingly, seven days later (July 31), SARS-CoV-2 RNA was detected from the designated quarantine dormitory. After inquiring whether the dorm was occupied, we learned two SARS-CoV-2 positive individuals were housed in the quarantine dorm site from July 26 to July 27.

No SARS-CoV-2 RNA was subsequently detected at any of the sites until August 19, the first day of class, when virus signal was detected from the 2 major Northern and Southern outflows (Figs 3 and 4). These data corresponded with the return of students for the start of the Fall semester, with student move-in to all dormitories, except the unoccupied overflow quarantine dorm, beginning on August 13 (Figs 3 and 4). Owing to the detection of SARS-CoV-2 RNA on August 19, we increased the frequency of sample collection from once/week to 3 times/week. Viral RNA was detected from the northern and southern outflows on August 24, (Fig 3). The wastewater emitted from most of the dormitories was obtained from the southern outflow site and this sampling site was positive for SARS-CoV-2 beginning on August 19^th^ and remained positive for SARS-CoV-2 throughout the remainder of the study (Figs 3 and 4). However, this outflow was collected from the quarantine dorm which may have led to the consistent positive signal. On August 26, SARS-CoV-2 was detected for the first time from the athletics outflow as well.

A cluster of 7 students was identified on August 31 in a dormitory from which effluent was collected in the northern outflow. Interestingly, the SARS-CoV-2 RNA signal was ~256- fold higher on August 31, the day after the announcement of the cluster, compared to the first positive reading on August 19 (Figs 3 and 4). From September 3 to September 25, 3 more clusters were announced in dormitories from which wastewater flows into the northern or southern outflows (Fig 2).

SARS-CoV-2 signal was first detected at the athletics wastewater collection site on August 26, one week after classes started. On October 5, a large cluster was announced from the University’s athletics program, which runs the majority of its operations in the athletic complex and nearby buildings. The wastewater from these athletics facilities buildings was collected from the athletics outflow site (Fig 1). As shown in Figs 3 and 4, one of the highest signals was obtained on October 5, the same day that the athletics cluster was announced.

Samples collected from the unoccupied dorm occasionally showed signal likely due to the extreme proximity of outflow pipes within the manhole to an occupied dorm. Another plausible hypothesis to explain this aberrant signal could be the presence of SARS CoV-2 positive workers in the building during the fall semester.

### Comparison of sample processing methods for detection of SARS-CoV-2 in wastewater

**Fig 5:**
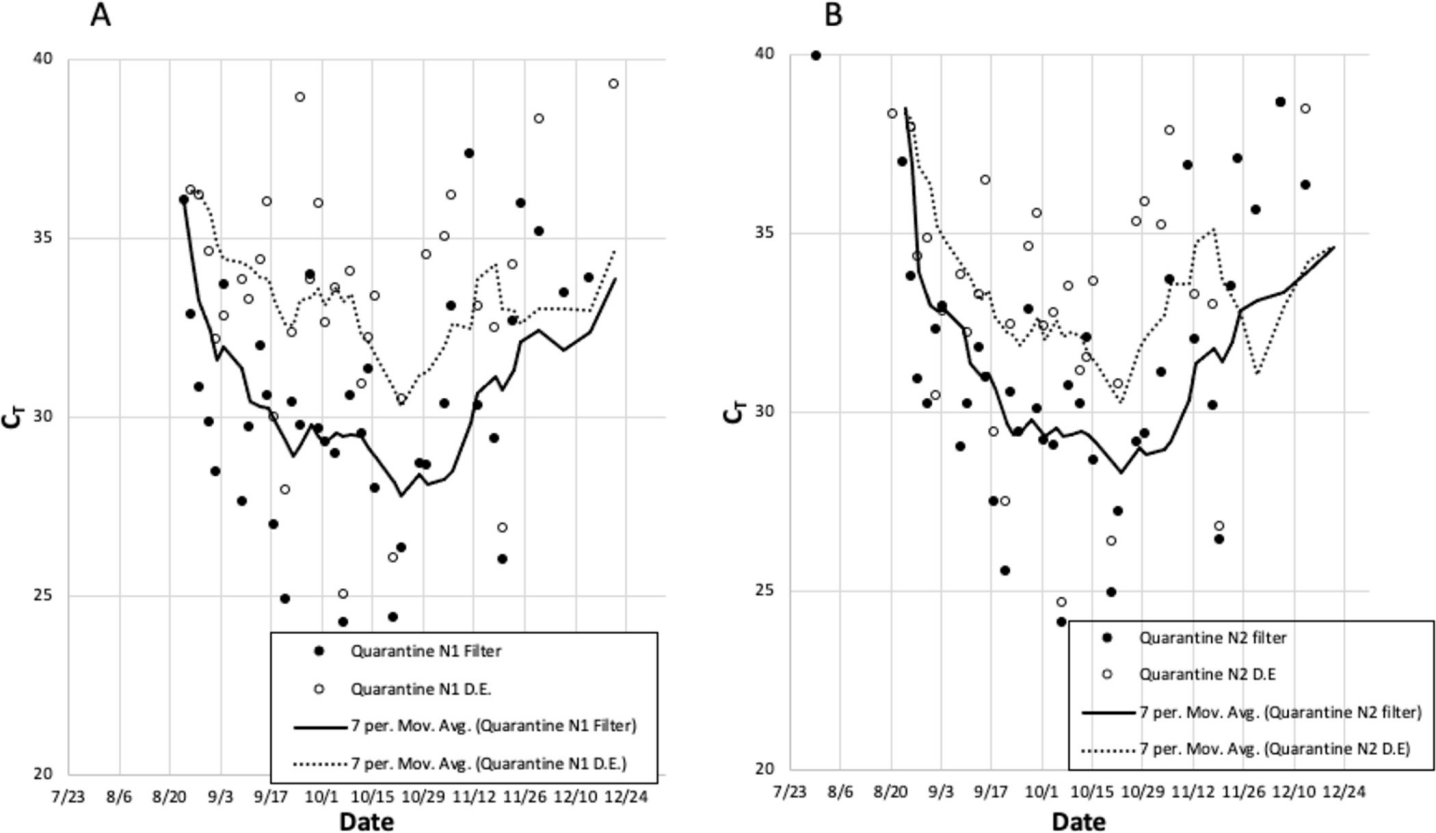
A comparison of direct extract and filtration methodologies with samples taken from the quarantine dorm. A shows Ct values with the N1 primer set and B shows Ct values with N2 primer set. Both figures use black dots for filter samples and open circles for direct extract samples.

A total of 220 individual wastewater samples were collected from the 5 UNCW sites from July 24 until December 21, 2020. After collecting the wastewater from the 5 locations on campus, each sample was heat inactivated and then processed using two separate methodologies as described in Materials and Methods. As shown in Fig 5, comparing the 7-day moving average of the Ct values obtained from the filters versus direct extracts suggested that, for the majority of samples, the filter Ct readings were lower than the DE samples. Ct values from the quarantine dorm that had two positive replicate values for each primer set, N1 and N2, were used to evaluate differences between the filtered and direct extract samples by comparing the differences between N1 or N2 values for the two sample preparation methods and testing the hypothesis that the average difference was zero. For N1, the mean difference in Ct values was -3.41, which was significantly different from zero by t-test (t value = -8.43, df = 20, p<0.0001), showing that filtered samples had significantly lower Ct values, i.e., higher SARS-CoV-2 signal, than direct extracts. Similarly, for N2 the mean difference was -2.53, which was significantly different from zero by t-test (t value = -6.29, 24 df, p<0.0001), showing again that the filtered samples yielded lower Ct values than the direct extracts.

### Comparison of N1 and N2 primer sets for detection of SARS-CoV-2 virus in wastewater samples

The patterns of Ct values for N1 and N2 appeared to be similar (Figs 3 and 4). To determine if there were significant differences between these two primer sets, we first analyzed only samples that had a positive result in both replicates for both N1 and N2. Calculation of the mean Ct value of N1 minus the mean Ct value of N2 for each sample resulted in an average value of -0.35. We then tested if the range of average difference included zero by Student’s t-test, finding that the value of zero was significantly different from the observed mean of -0.35 (t = -3.09, df = 93, p<0.01), showing that N1 values were significantly lower than N2 values. These results suggested that the N1 primer set was more sensitive than the N2 primer set, as lower Ct values denote higher expression.

We then tested the hypothesis that differences between average N1 and average N2 Ct values for each sample were a function of average Ct value using linear regression (N1 minus N2 value vs. corresponding overall average) to determine if lower Ct values yielded smaller N1-N2 values or vice versa. The analysis yielded no significant regression–i.e., values for N1-N2 were NOT a function of the average Ct value (F = 0.05, p = 0.81, df = 1,92). A comparison of Ct values among sample locations using one-way ANOVA revealed a significant effect (F = 12.8, df = 3,89, p<0.0001). A comparison of means using the Tukey-Kramer HSD *a posteriori* test showed that the Quarantine location had a significantly lower Ct value (average = 30.4) compared to the other three locations [all access points], where average Ct values were 33.4–33.8 and not significantly different from each other. The one way ANOVA found no significant effect (F = 2.22, df = 3,89, p = 0.091) in determining effect of location on N1 minus N2 Ct values.

### Individual SARS-CoV-2 positive cases and bed usage in the quarantine dorm

**Fig 6:**
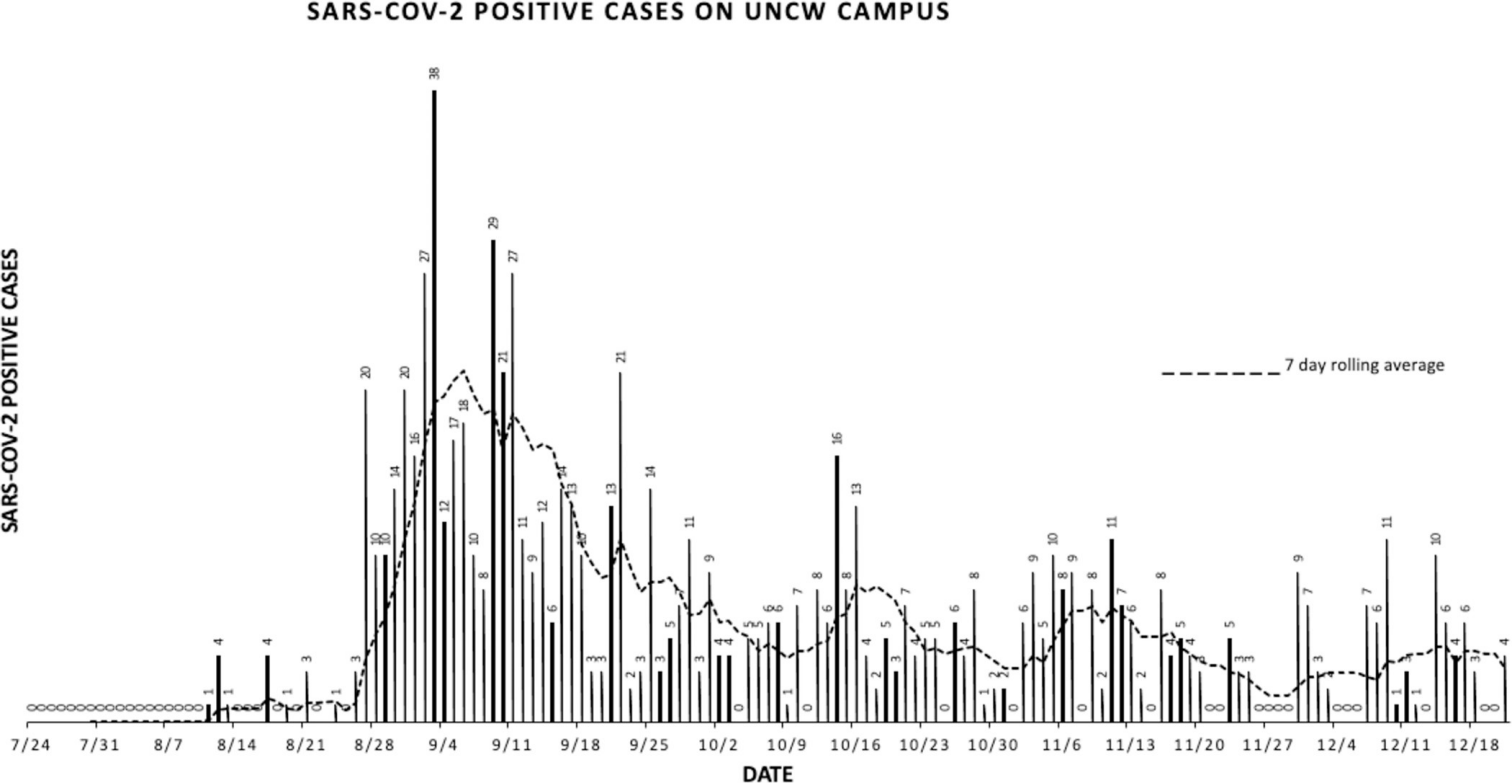
Graphical representation of SARS CoV-2 cases on UNCW’s campus during the fall semester 2020 with 7- day rolling average.

There was a spike in the number of individuals that tested positive for SARS-CoV-2 at the beginning of the Fall semester with the peak 7 day moving average occurring September 4^th^ (Fig 6). The first individual SARS-CoV-2 positive test was recorded on August 11, 8 days before the start of classes. Cases continued to climb in late August and early September reaching a peak of 38 positive cases on September 3. On September 8, 2020 based on guidance from the North Carolina Department of Health and Human Services (NCDHHS), the administration of UNCW implemented a de-densification strategy in an effort to reduce transmission of the virus and the number of positive cases. Of the 3,685 students residing on campus at the time only 1,900 of those students were living in single occupancy housing with the rest living in traditional collective dorms. After a massive spike in cases, UNCW was mandated by the New Hanover County Health Department on September 8, 2020, to “de-densify” the on-campus population by making all on- campus housing single occupancy. After the de-densification, the on campus population decreased to 2,975 with 99% of students living in single spaces. After this plan was initiated, case numbers declined through the remainder of September with only 3 cases recorded on September 30 suggesting that de-densifying student living had a positive impact on preventing the spread of the pathogen. SARS-CoV-2 positive cases rose again in mid-October peaking on October 14 when 16 cases were recorded. After this mid-October peak, daily case numbers ranged from 0 to 13 for the remainder of the Fall semester.

**Fig 7:**
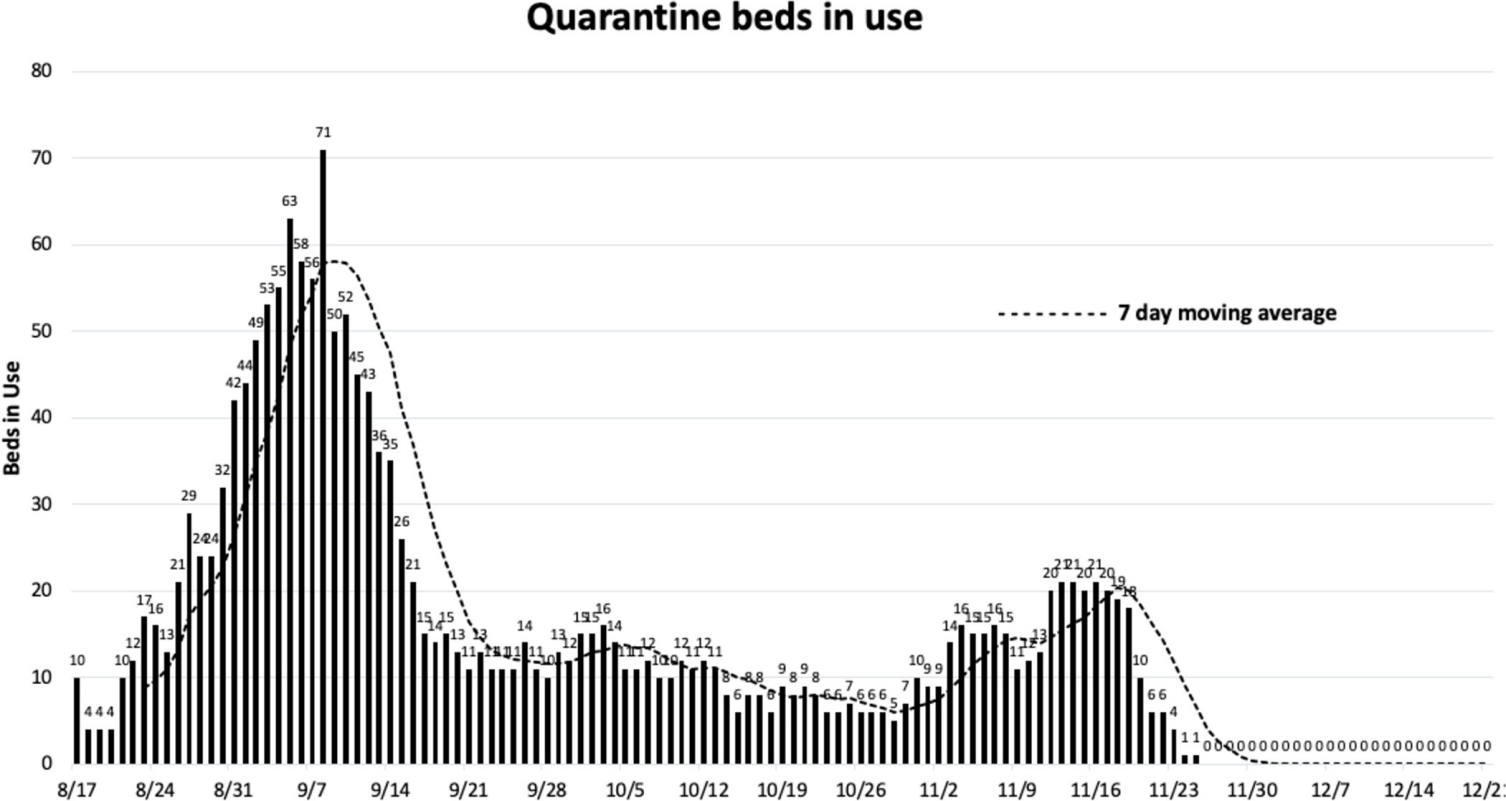
Quarantine dorm beds in use during fall semester 2020.

Another measure of the prevalence of SARS-CoV-2 in the student population was the number of beds in use in the quarantine dorm which can hold a maximum of 150 quarantined persons. As shown in Fig 7, six individuals were quarantined in the dormitory on August 11 and this number steadily rose through August into September with the number of quarantined individuals reaching its highest number on September 8, when 71 individuals were housed in the dormitory. After this peak, the number of quarantined persons dropped to twelve on September 30. From that point on, the number of quarantined persons per day ranged from 1–21 peaking at 21 on November 16. Due to Covid-19, the Fall semester was truncated with classes and final exams being moved to an online format after the Thanksgiving break, and the majority of students were no longer able to reside on campus following the break. The early end to the semester led to a reduced daily case count (Fig 6) and no individuals were quarantined from November 27 through the end of our study in mid-December (Fig 7).

## Discussion

The University of North Carolina Wilmington’s (UNCW) sewer shed is a closed loop system allowing for a direct approach to sampling of wastewater without worry of dilution from rainwater termed Inflow and Infiltration (I&I), which could make it difficult to accurately discern what amount of SARS-CoV-2 RNA originated from outside the campus system. The closed nature of the UNCW campus wastewater system, combined with strategic sampling sites allowed for sources of infection to be easily identified and, most importantly, localized to a particular region of campus. Previous work in other near to source testing operations were able to detect a single individual infected with SARS CoV-2 [17] Individual testing of students, faculty, and staff was not compulsory during the Fall 2020 semester when we conducted our wastewater testing and individual tests were only performed at the request of the individual. If, at any point, a “cluster” of infection was identified, the campus body was notified. A cluster was defined as five or more positive cases that are considered in close proximity by location in the last 14 days.

The use of WBE for SARS CoV-2 and a wide array of other agents, has been consistently shown to be an extremely useful tool for detecting pathogens and monitoring infection [18]. WBE allows for population level SARS-CoV-2 surveillance as opposed to monitoring the disease at an individual scale [14, 17, 18]. An additional benefit of SARS-CoV-2 WBE is the anonymity of the testing. Healthcare privacy laws regarding testing of the individual are not applicable for this macroscopic level of testing [18]. As individuals cannot opt out of this anonymous large-scale testing, the data obtained from WBE screening provides a more complete picture of SARS-CoV-2 prevalence at the population level. In addition, especially at the onset of a disease outbreak, frequent testing of wastewater can enable detection of SARS CoV-2 before individual tests reveal the presence of the virus. The early detection of virus from pooled, anonymous samples can provide public health officials with critical information and these data may be used to help inform public health policies, testing allocation, decision making, and the implementation of strategies to mitigate the spread of SARS-CoV-2 [19]. At the beginning of our study two methodologies were compared for detection of SARS-CoV-2 in wastewater to determine best methodology in terms of efficiency and sensitivity. We found with both N1 and N2 primer sets respectively, the sensitivity of the membrane filters was in most cases, one log greater than the direct extracts. It is likely due to the concentration of sample given that the direct extracts were only 600 μL while the membrane filters had, at times, 100 mL of wastewater passed through it. While the sensitivity was lower the efficiency was higher in terms of time to results, and the majority of the direct extract samples also were positive for SARS CoV-2 RNA.

WBE utilized on UNCW’s campus during the Fall 2020 semester was able to give not only the most accurate indication of active infections on campus in comparison to individual testing, but also allowed for the advance detection of infection clusters prior to individual positive Covid-19 results (Figs 2 & 3). In assessing the presence of SARS-CoV-2 in the samples, two primer sets were used which bound to two distinct but relatively proximal regions of the nucleocapsid gene. A positive signal with just one of these primer sets with qPCR was enough to call the sample positive, however we sought to determine the frequency at which both primers hit on samples and our results showed that the N1 primers detected target sequence at a greater frequency than N2. Using only Ct data from samples that had 2 replicates for N1 and N2, the mean value of N1-N2 was -0.35; a value of zero was significantly different than the observed mean (t = -3.09, df = 93, p<0.01). Our data are corroborated by Vogels *et al*. who found similar results from an analysis of 172 nasopharyngeal samples [20]. This slight difference between N1 and N2 primer sets has been corroborated in other studies [21]. Together, the consistently higher sensitivity of filters vs direct extracts combined with the efficiency of filtration make a strong case that filtration is a better method for the detection of SARS CoV-2 in wastewater. Direct extract methodology could still be useful for lower tech monitoring of wastewater as it is still able to detect some RNA [22].

At the end of the semester, SARS CoV-2 RNA was still detected after the quarantine dorm had been empty for a week. A flush of the dormitory plumbing was performed. In total, approximately 414 gallons of only water was flushed through the system, and subsequent RT-qPCR testing showed no detectable SARS CoV-2 RNA. The persistence of RNA was likely due to the old construction of the dormitory (1957) and connecting plumbing which is made from terra-cotta as opposed to the more modern PVC. Medina *et al* describes their groups findings of antibiotic resistant bacteria in the insides of terra cotta sewer pipes [23]. Due to its porous nature, we hypothesize that more RNA is able to adhere to the inside of the pipe as opposed to the more modern plumbing options. It is possible SARS CoV-2 RNA would have persisted longer if the flush was not performed. A study conducted recently in China after a city was declared “COVID free” determined that SARS CoV-2 RNA persisted in wastewater for 8 days at 26°C [24]. Another study by Bivins *et al* showed very similar results [25].

Our study of wastewater on a closed loop university campus has demonstrated that WBE is an extremely useful tool for tracking and predicting cases of infection of SARS CoV-2. The lack of influx of external contaminants into the system and user ability to conduct individual building tests makes this an ideal system for WBE. During the fall 2020 semester, cases were able to be accurately predicted and measured using five sites on campus. We determined that membrane filtration was the optimal method of wastewater concentration for molecular testing and showed that while there was a statistical difference in the N1 & N2 primer sets, both sets still are reliable indicators of SARSCoV-2 RNA. Future work will include building level surveillance to get a more accurate and specific picture of the state of the pandemic on campus. Additionally, the emergence of variants begs the question of whether variants can also be tracked using WBE.

## Supporting information

S1 Data Campus wastewater data. Ct values for each sampled site from July 31, 2020 to December 7, 2020.
(XLSX)

S2 Data Number of SARS CoV-2 positive cases on the UNCW campus from July 24, 2020 to December 21, 2020. (XLSX)

S3 Data Number of individuals in the quarantine dormitory from August 11, 2020 to December 21, 2020. (XLSX)
